# Prediction value of cystatin C for postoperative acute kidney injury of acute type A aortic dissection

**DOI:** 10.5937/jomb0-56220

**Published:** 2026-01-28

**Authors:** Shouming Li, Xin Zhao, Zhenhua Wang, Han Song, Yongmin Liu

**Affiliations:** 1 Beijing Anzhen Hospital, Capital Medical University, Department of Cardiovascular Surgery, Beijing, China; 2 Qilu Hospital of Shandong University, Department of Cardiovascular Surgery, Jinan, Shandong Province, China

**Keywords:** acute type A aortic dissection, acute kidney injury, prediction model, akutna disekcija aorte tipa A, akutna bubrežna insuficijencija, prediktivni model

## Abstract

**Background:**

This study aimed to develop an early prediction model for postoperative acute kidney injury (AKI) in patients with acute type A aortic dissection (ATAAD) undergoing total a rch replacement (TAR) combined with frozen elephant trunk (FET) implantation. Specifically, it investigated the association between preoperative cystatin C levels and postoperative AKI while incorporating other relevant risk factors into the analysis.

**Methods:**

A single-centre case-control study was conducted, including 202 patients treated between January 2018 and December 2019. Patients were divided into an AKI group (n=73) and a non-AKI group (n = 129) based on postoperative renal outcomes.

**Results:**

Univariate analysis revealed that a preoperative history of hypertension (P = 0.013), white blood cell (WBC) count (P&lt; 0.001), serum creatinine (P &lt; 0.001), blood urea nitrogen (P &lt; 0.001), and cystatin C (P&lt; 0.001) were significantly associated with postoperative AKI. Postoperative variables, including duration of mechanical ventilation (P&lt; 0.001), length of ICU stay (P&lt; 0.001), acute respiratory failure (P = 0 .0 1 8 ), acute cerebrovascular events (P= 0.042), and 30-day mortality (P&lt; 0.001) also differed significantly between groups. Multivariate logistic regression incorporating preoperative variables identified cystatin C (OR: 11.541, 95% CI: 3.811 -34.953 , P&lt; 0.001), WBC count (OR: 1.122, 95% CI: 1.013-1.242, P= 0.028), and history of hypertension (OR: 3.080, 95% CI: 1.188-7.990) as independent risk factors for postoperative AKI. Survival analysis further confirmed a significant association between preoperative cystatin C levels and the incidence of AKI in both crude and subgroup analyses. A clinical prediction model was developed based on the multivariate results. Internal validation showed a concordance index (C-index) of 0.804 for the training set and 0.609 for the validation set.

**Conclusions:**

Preoperative cystatin C level was identified as an independent predictor of postoperative AKI. Incorporating cystatin C with other preoperative clinical risk factors may enhance the predictive accuracy for postoperative AKI in patients with ATAAD undergoing total arch replacement with FET implantation.

## Abstract (sr)

**Uvod:**

Ova studija imala je za cilj da razvije model za ranu predikciju postoperativne akutne bubrežne insuficijencije (AKI) kod pacijenata sa akutnom disekcijom aorte tipa A (ATAAD) koji su podvrgnuti totalnoj zameni aortnog luka (TAR) u kombinaciji sa FET (frozen elephant trunk - tehni-ka kombinovane rekonstrukcije aortnog luka i silazne torakalne aorte uz upotrebu zamrznutog grafta). Poseban fokus je bio na ispitivanju povezanosti između preoperativnih nivoa cistatina C i pojave postoperativne AKI, uz uključivanje drugih relevantnih faktora rizika u analizu.

**Metode:**

Sprovedena je retrospektivna studija slučaj-kontrola u jednoj ustanovi, koja je obuhvatila 202 pacijenta lečena u periodu od januara 2018. do decembra 2019. godine. Na osnovu postoperativne funkcije bubrega, pacijenti su podeljeni u AKI grupu (n=73) i grupu bez AKI (n=129).

**Rezultati:**

Univarijantna analiza je pokazala da su preoperativna anamneza hipertenzije (P= 0.013), broj leukocita (WBC) (P&lt; 0.001), serumski kreatinin (P&lt; 0.001), urea u krvi (P&lt; 0.001) i cistatin C (P&lt; 0.001) značajno povezani sa pojavom postoperativne AKI. Postoperativne varijable kao što su trajanje mehaničke ventilacije (P&lt; 0.001), dužina boravka na intenzivnoj nezi (P&lt; 0.001), akutna respiratorna insuficijencija (P= 0.018), akutni cerebrovaskularni događaji (P= 0.042) i 30-dnevna smrtnost (P&lt; 0.001) takođe su se značajno razlikovale između grupa. Multivarijantna logistička regresiona analiza preoperativnih faktora identifikovala je cistatin C (OR: 11.541, 95% CI: 3.811-34.953, P = 0.001), broj leukocita (OR: 1.122, 95% CI: 1.013-1.242, P= 0.028) i istoriju hipertenzije (OR: 3.080, 95% CI: 1.188-7.990, P= 0.021) kao nezavisne faktore rizika za razvoj postoperativne AKI. Analiza preživljavanja je dodatno potvrdila značajnu povezanost između preoperativnog nivoa cistatina C i učestalosti AKI, kako u osnovnoj, tako i u podgrupnoj analizi. Na osnovu rezultata multivarijantne analize razvijen je klinički prediktivni model. Interna validacija pokazala je indeks slaganja (C-indeks) od 0.804 za trening skup i 0.609 za validacioni skup.

**Zaključak:**

Preoperativni nivo cistatina C identifikovan je kao nezavisan prediktor postoperativne AKI. Uključivanje cistatina C zajedno sa drugim preoperativnim kliničkim faktorima rizika može da pospeši tačnost predikcije AKI kod pacijenata sa ATAAD koji se podvrgavaju totalnoj zameni luka aorte sa FET implantacijom.

## Introduction

Acute type A aortic dissection (ATAAD) is a life-threatening condition that often involves multiple organ systems, with the kidneys being among the most frequently affected. A subset of ATAAD patients requires aortic arch surgery, a complex procedure typically involving hypothermic circulatory arrest, which can further compromise renal perfusion and exacerbate kidney injury. Acute kidney injury (AKI) is a common complication in this population, with reported incidence rates ranging from 23% to 42.6% across various studies [1] [2]. AKI in ATAAD patients poses significant clinical concerns, contributing to increased morbidity, prolonged hospital stays, and, in some cases, markedly poorer long-term survival outcomes [3]. The management of severe AKI, particularly stage 3, remains challenging due to limited treatment options. Currently, continuous renal replacement therapy (CRRT) is the primary intervention for stage 3 AKI. Although potentially life-saving, CRRT is associated with high costs and substantial resource demands [4], underscoring the need for improved early detection and prevention strategies.

The Kidney Disease: Improving Global Outcomes (KDIGO) criteria are widely accepted as the gold standard for AKI diagnosis [5]. However, traditional biomarkers such as serum creatinine and urine output have limitations, particularly in detecting early or subclinical kidney injury. Delays in creatinine elevation or variability in urine output can result in missed or late diagnoses. Consequently, there is increasing interest in novel biomarkers that can provide more sensitive and timely detection of AKI [6] [7] [8]. Among these, serum cystatin C has emerged as a promising candidate, with evidence suggesting it may outperform conventional markers in early AKI prediction. Nonetheless, studies specifically evaluating the utility of cystatin C in the context of ATAAD and its association with postoperative AKI remain limited.

Therefore, this study aims to investigate the association between preoperative serum cystatin C levels and the development of postoperative AKI in patients with ATAAD undergoing total aortic arch replacement (TAR) combined with frozen elephant trunk (FET) stent implantation. By identifying potential predictive value, the goal is to construct a clinically applicable risk model to facilitate the early recognition of high-risk patients, enable timely interventions, and potentially improve patient outcomes.

## Materials and methods

### Study design

This retrospective case-control study was registered at ClinicalTrials.gov (ID: NCT05039814) and approved by the institutional ethics committee (Approval No. KYLL-202008-017). Informed oral consent was obtained from all participants in accordance with the ethical standards outlined in the Declaration of Helsinki.

### Patient selection

Consecutive patients diagnosed with ATAAD via computed tomography angiography (CTA) and treated at a single cardiac centre between 2018 and 2019 were enrolled. Inclusion criteria were: (1) age between 18 and 80 years and (2) a confirmed diagnosis of ATAAD based on CTA findings. Exclusion criteria included (1) pre-existing chronic kidney disease, (2) prior history of cardiovascular surgery, and (3) pregnancy. A total of 202 patients met the eligibility criteria. It was stratified into two groups based on the presence or absence of postoperative AKI: the Non-AKI group (n = 129) and the AKI group (n = 73).

### Surgical procedure

All patients underwent TAR combined with FET implantation. Hypothermic circulatory arrest was maintained at 26°C, with cerebral protection achieved via unilateral low-flow antegrade perfusion. All procedures were performed by three senior cardiac surgeons, each with an annual caseload exceeding 200 surgeries. Detailed surgical techniques have been described in previous publications [9] [10].

### Definitions

The primary outcome was the incidence of post-operative AKI within 30 days following surgery, defined according to the KDIGO criteria. Secondary outcomes included all-cause mortality within the same period. Renal artery involvement (RAI) was defined as dissection extension into one or both renal arteries. Postoperative acute cerebrovascular events (ACVE) were defined as new-onset disturbances of consciousness confirmed by imaging within 30 days. Acute respiratory failure (ARF) was diagnosed as hypoxemia (PaO_2_<60 mmHg) and/or hypercapnia (PaCO_2_ > 50 mmHg) in the absence of supplemental oxygen, also within 30 days postoperatively.

### Laboratory measurements

Blood samples were collected routinely within 24 hours of admission and daily after surgery, then sent to the clinical biochemistry laboratory for analysis. Serum cystatin C levels were measured using a latex-enhanced immunoturbidimetric assay. This method involves the formation of immune complexes between cystatin C in the serum and anti-cystatin C antibodies in the reagent. A precipitating enhancer facilitates the aggregation of these complexes, producing turbidity. Under antibody excess and stable reaction conditions, the turbidity is proportional to the cystatin C concentration. Absorbance was measured at a wavelength of 570 nm to quantify cystatin C levels accurately.

### Statistical analysis

All analyses were performed using SPSS software (version 26.0; SPSS Inc., Chicago, IL, USA) and R software (version 4.1.0). Missing values were imputed using the Random Forest algorithm. The normality of continuous variables was assessed using the Shapiro-Wilk test and Q-Q plots. Normally distributed variables were expressed as mean ± standard deviation (SD) and compared using Student's t-test, while non-normally distributed variables were reported as median (Q1, Q3) and compared using the Wilcoxon rank-sum test. Categorical variables were presented as frequencies and percentages, with group comparisons performed using the chi-square test or Fisher's exact test, as appropriate.

Multivariable logistic regression analysis was conducted to identify independent risk factors for postoperative AKI. AKI-free survival was estimated using the Kaplan-Meier method, with differences between groups assessed using the log-rank test.

The dataset was randomly divided into training (70%) and validation (30%) sets. Feature selection was conducted using least absolute shrinkage and selection operator (LASSO) regression to determine the optimal penalty parameter (λ) that minimized misclassification. Variables with non-zero coefficients were retained for model development using logistic regression on the training set.

Model discrimination was evaluated using the area under the receiver operating characteristic curve (AUC), while calibration was assessed via calibration plots in the validation dataset. Decision curve analysis (DCA) was performed to evaluate the clinical utility of the model. The final model was deployed as an interactive web-based tool, available at https://auguster.shinyapps.io/DynNomapp/, hosted by Shinyapps.io. A two-sided p-value<0.05 was considered statistically significant.

## Results

### Baseline characters

There were statistically significant differences in the history of hypertension (HT), white blood cell count (WBC), serum creatinine (SCr), blood urea nitrogen (BUN), and cystatin C levels between the two groups. In contrast, renal artery involvement (RAI) was not significantly associated with postoperative AKI (Table 1).

**Table 1:** Baseline characters. BUN: Blood Urea Nitrogen; CHD: Coronary Artery Disease; CTnI: Cardiac Troponin I; DD: D-Dimer; DM: Diabetes Mellitus; HT: Hypertension; HGB: Hemoglobin; PLT: Platelet; RAI: Renal Artery Involvement; RBC: Red Blood Counts; SCr: Serum Creatinine; Cys-c: Cystatin C; WBC: White Blood Cell Counts.

Variables	Non-AKI (n = 129)	AKI (n=73)	Total (n=202)	P
Male	93 (72.1)	60 (82.2)	153 (75.7)	0.108
Age (y)	52.02±11.59	52.23±11.93	52.10±11.69	0.903
Smoke	61 (47.3)	38 (52.1)	99 (49.0)	0.515
Drink	71 (55.0)	43 (58.9)	114 (56.4)	0.595
HT	96 (74.4)	65 (89.0)	161 (79.7)	0.013
CHD	28 (21.7)	9 (12.3)	37 (18.3)	0.098
DM	4 (3.1)	1 (1.4)	5 (2.5)	0.772
WBC (10^9^/L)	9.86 (8.10, 12.06)	11.86 (9.28, 13.73)	10.37 (8.65, 12.62)	<0.001
RBC (10^12^/L)	4.01±0.51	4.11±0.57	4.04±0.54	0.222
HGB (g/L)	126.00 (112.00, 136.00)	127.00 (114.07, 141.00)	126.00 (113.50, 138.00)	0.398
PLT (10^9^/L)	161.00 (132.00, 196.50)	152.00 (126.00, 192.50)	155.32 (131.00, 193.00)	0.444
DD (μg/mL)	2.75 (1.24, 5.98)	3.29 (1.25, 5.93)	3.06 (1.29, 5.93)	0.870
CTnI (ng/L)	36.80 (4.15, 276.61)	73.93 (5.43, 218.38)	46.30 (4.53, 241.00)	0.368
BUN (mmol/L)	7.03 (4.95, 9.07)	8.40 (6.75, 11.63)	7.63 (5.67, 9.74)	<0.001
SCr (μmol/L)	76.00 (63.00, 94.44)	107.00 (81.00, 142.50)	84.00 (65.00, 107.00)	<0.001
Cys-C (mg/L)	0.88 (0.72, 1.05)	1.12 (0.93, 1.58)	0.95 (0.76, 1.16)	<0.001
RAI	117 (90.7)	63 (86.3)	180 (89.1)	0.335

### Perioperative results

Surgical duration, extracorporeal circulation (ECC) time, cerebral perfusion strategy, and red blood cell infusion (RBCI) were not significantly associated with postoperative AKI. In contrast, mechanical ventilation time (MVT), length of ICU stay, postoperative ARF, ACVE, and early mortality (defined as all-cause death within 30 days post-surgery) were significantly associated with the occurrence of postoperative AKI (Table 2).

**Table 2:** Perioperative results. ACVE: acute cerebrovascular event; ARF: acute respiratory failure; BCP: bilateral cerebral perfusion; ECC: extracorporeal circulation; MVT: mechanical ventilation time; RBCI: red blood cell infusion; SCP: selective cerebral perfusion.

Variables	Non-AKI (n = 129)	AKI (n=73)	Total (n=202)	P
Surgery time (min)	490.00 (440.00, 552.50)	480.00 (422.50, 575.00)	490.00 (430.00, 556.25)	0.632
ECC time (min)	225.00 (202.50, 263.50)	213.00 (189.00, 262.50)	220.50 (197.00, 261.75)	0.214
Clamp time (min)	144.00 (129.00, 166.00)	133.00 (116.00, 164.00)	141.00 (124.00, 165.25)	0.120
SCP time (min)	26.00 (19.00, 34.00)	29.00 (21.00, 35.00)	27.00 (20.00, 34.00)	0.298
BCP	95 (73.6)	51 (69.9)	146 (72.3)	0.564
RBCI (U)	4.00 (2.00, 4.00)	4.00 (2.00, 4.00)	4.00 (2.00, 4.00)	0.276
MVT (h)	32.00 (17.00, 61.00)	67.00 (42.50, 117.50)	42.00 (20.00, 77.75)	<0.001
ICU time (h)	180.00 (129.50, 225.00)	234.00 (162.00, 324.00)	188.00 (135.50, 250.25)	<0.001
ARF	72 (55.8)	53 (72.6)	125 (61.9)	0.018
ACVE	7 (5.4)	10 (13.7)	17 (8.4)	0.042
Reoperation	1 (0.8)	1 (1.4)	2 (1.0)	>0.990
Death	12 (9.3)	29 (39.7)	41 (20.3)	<0.001

### Multivariable analysis

The results indicated that HT, WBC, and cystatin C levels were significantly associated with postoperative AKI, suggesting that these variables may serve as independent risk factors (Table 3).

**Table 3:** Multivariable analysis. Cys-c: cystatin C; HT: hypertension; WBC: white blood cell counts.

Variables	B	Wald	P	OR	95% CI for OR	
					LL	UL
Constant	-5.318	30.471	<0.001	0.005	-	-
HT	1.125	5.353	0.021	3.080	1.188	7.990
WBC (10^9^/L)	0.115	4.857	0.028	1.122	1.013	1.242
Cys-C (mg/L)	2.446	18.717	<0.001	11.541	3.811	34.953

### Survival analysis and subgroup analysis

**Figure 1:**
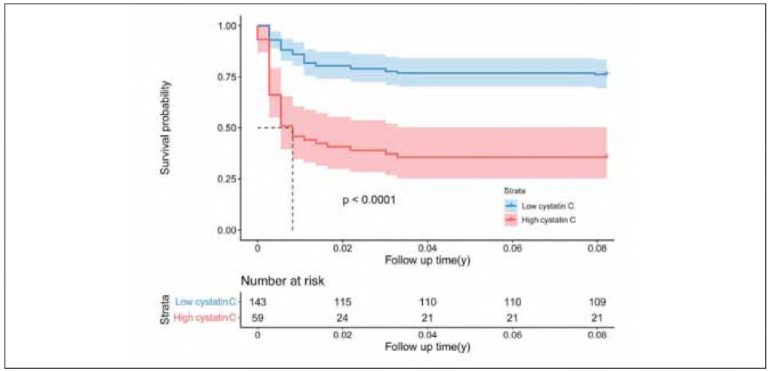
Freedom from postoperative acute kidney injury.<br>95% confidence intervals were shown as shadings.

**Figure 2:**
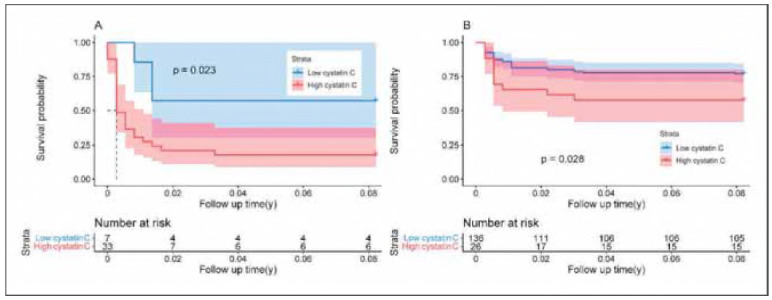
Freedom from postoperative acute kidney injury,<br>95% confidence intervals were shown as shadings. A: Low serum creatinine group; B: High serum creatinine group.

Patients were stratified into two subgroups based on whether their cystatin C level exceeded 1.110 mg/L. Survival analysis demonstrated a statistically significant association between elevated cystatin C levels and the occurrence of postoperative AKI (Figure 1). To assess the robustness of this association, a subgroup analysis was performed by dividing patients according to SCr levels, using 115 mmol/L as the cutoff. The results confirmed that cystatin C remained significantly associated with postoperative AKI in both SCr subgroups (Figure 2).

### Prediction model

**Figure 3:**
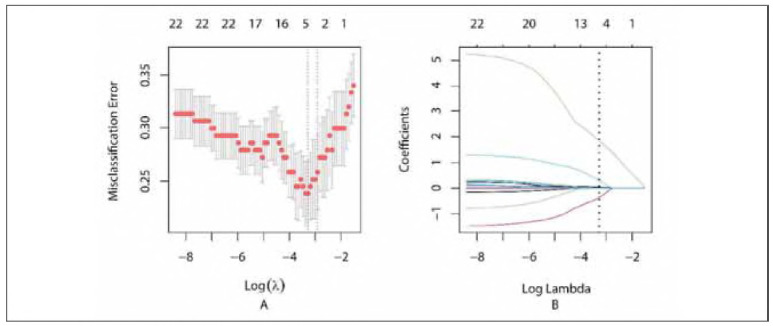
(Supplementary) Summary of Lasso regression.

**Figure 4:**
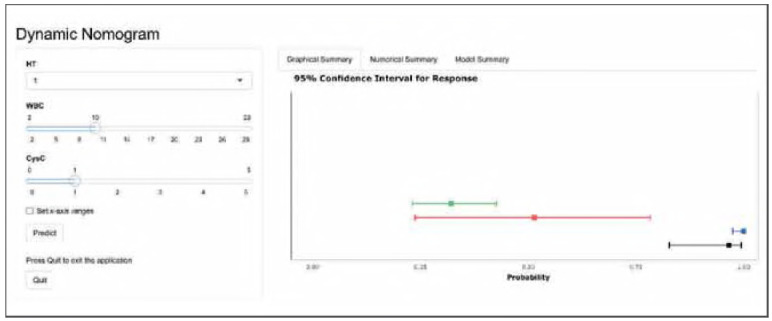
(Supplementary) The web calculator.<br>Cys-c: cystatin C; HT: hypertension; WBC: white blood cell counts

**Figure 5:**
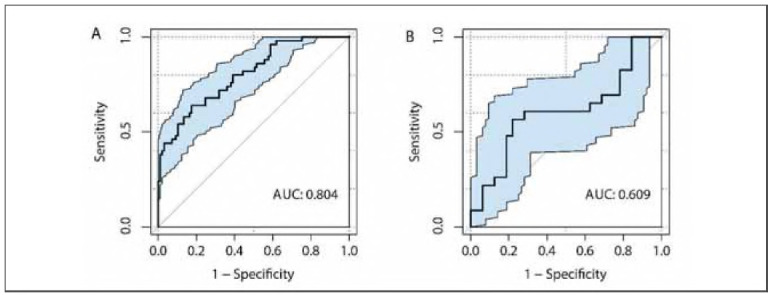
Receiver operating characteristic curves.<br>A: Training set; B: Validation set.

**Figure 6:**
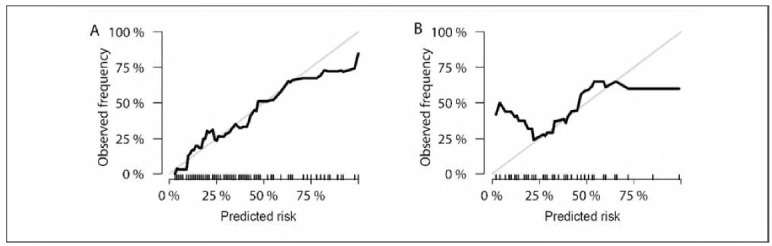
Calibration plot.<br>A: Training set; B: Validation set.

**Figure 7:**
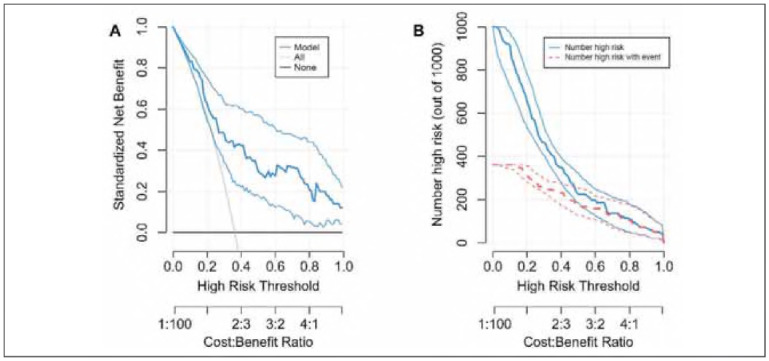
(Supplementary) Decision curve analysis of the prediction.<br>A: Decision curve; B: Clinical impact curve.

The optimal λ value identified by Lasso regression was 0.03765697 (Figure 3, Supplemental Material). A summary of the final predictive model is presented in Table 4 (Supplemental Material). The model has been deployed as an interactive web-based tool and is accessible at https://auguster.shinyapps.io/DynNomapp/, as illustrated in Figure 4 (Supplemental Material). Model performance in terms of discrimination and calibration is shown in Figure 5 and Figure 6, respectively. The results of the DCA are displayed in Figure 7 (Supplemental Material).

**Table 4:** (Supplementary) Summary of the prediction model. Cys-c: cystatin C; HT: hypertension; WBC: white blood cell counts.

Variables	B	Std. Error	*P*
Constant	-5.802	1.172	<0.001
HT	1.003	0.596	0.092
WBC (10^9^/L)	0.081	0.062	0.192
Cys-C (mg/L)	3.247	0.731	<0.001

## Discussion

The KDIGO criteria, which rely on both SCr and urine volume, have inherent limitations. SCr levels are influenced by muscle mass and body weight, while urine volume is affected by circulatory blood volume and diuretic use, both of which are common in cardiovascular surgery. These factors complicate the timely and accurate diagnosis of AKI. As a result, there is growing interest in exploring new biomarkers and clinical models to enhance detection.

Several promising biomarkers have emerged in recent years, showing potential for early AKI detection, prognosis prediction, and guiding therapeutic interventions. However, their widespread clinical adoption has been hindered by variability in performance across patient populations, limited availability, and high costs. Cystatin C has gained recognition as a highly promising biomarker for AKI. It is synthesized by all nucleated cells and freely filtered by the glomerulus, where it is reabsorbed and metabolized by renal tubules without being secreted back into the bloodstream. Consequently, serum cystatin C levels rise when the glomerular filtration rate (GFR) declines.

Furthermore, cystatin C levels are not significantly influenced by common variables such as gender, age, and race and remain stable under standard storage conditions. The detection method for cystatin C is well-established and has been shown to outperform SCr in assessing GFR. Notably, cystatin C levels rise earlier than SCr levels during AKI [11] [12], which can be attributed to molecular differences. The molecular weight of cystatin C (13 kDa) is larger than that of SCr (113 Da). In the early stages of AKI, alterations in the filtration membrane hinder cystatin C passage, while smaller creatinine molecules pass through more easily [13].

Numerous studies have demonstrated the diagnostic and predictive value of cystatin C for AKI [14] [15]. However, a universally accepted threshold for diagnosing AKI has not yet been established, and research focusing specifically on ATAAD patients undergoing TAR and FET implantation remains limited. The present study established a correlation between preoperative cystatin C levels and the occurrence of postoperative AKI in ATAAD patients, underscoring its potential as a predictive biomarker in this population.

A single biomarker often falls short of providing accurate predictions in clinical settings. Therefore, prediction models integrating both biomarkers and clinical risk factors are gaining traction, as they can enhance both the sensitivity and specificity of predictions. Essential hypertension is a leading cause of ATAAD, and chronic hypertension can lead to renal complications such as glomerular fibrosis, renal atrophy, arteriosclerosis, and even renal parenchymal ischemia, which impair kidney function [16] [17]. Moreover, during ATAAD, inflammatory cells aggregate at the lesion site, initiating a cascade of inflammatory responses. These cells infiltrate the lesion microenvironment, exacerbating damage and increasing the risk of postoperative kidney injury [18] [19]. Some studies have suggested that markers related to WBC counts could serve as valuable early indicators for AKI, further highlighting the role of inflammation in predicting renal complications [20].

These factors, along with preoperative renal malperfusion, hemolysis due to prolonged cardiopulmonary bypass, and ischemia-reperfusion injury, contribute to the high incidence of postoperative AKI in ATAAD patients [4]. Our study found no significant correlation between RAI or ECC time and postoperative AKI, which may reflect advancements in surgical techniques that have reduced ECC duration. Importantly, the kidney may maintain blood supply through the false lumen, mitigating some of the effects of reduced perfusion. However, the effects of preoperative renal artery dissection and its impact on renal perfusion remain unclear and warrant further investigation. Renal malperfusion is the primary factor leading to postoperative AKI, and renal perfusion status during the perioperative period is a critical determinant of postoperative renal function. In cases of renal artery involvement due to aortic dissection, poor renal perfusion can manifest as dynamic or static ischemia. Dynamic ischemia typically improves following central aortic repair, while static ischemia remains largely unchanged. The risk of postoperative AKI is related not only to the pattern of renal artery involvement but also to the duration of ischemia. Unfortunately, due to practical limitations, continuous monitoring and assessment of renal perfusion from the onset of the disease to surgery is often unfeasible. Aortic CTA is commonly used to evaluate preoperative renal artery involvement, but its lack of dynamic imaging and quantitative measures limits its ability to assess renal perfusion accurately. Thus, new diagnostic methods or biomarkers with greater sensitivity and specificity are needed to improve preoperative risk stratification.

Previous models for predicting AKI have typically focused on identifying patients who may require CRRT However, even a slight rise in SCr can have significant long-term effects on patient prognosis [21]. Given that renal malperfusion often underlies AKI in ATAAD patients, early prediction of prerenal AKI is critical. Early detection allows for timely interventions that may prevent the progression of renal injury and improve patient outcomes. AKI is associated with significant organ damage, and when combined with the stress of surgery, it can further deteriorate a patient's clinical status.

Our results demonstrated that postoperative AKI was closely linked with severe complications, including ACVE, ARF, and even mortality during hospitalization. This emphasizes the critical importance of early identification and management of AKI. In this study, patients were categorized into AKI and non-AKI groups rather than subdividing by severity, which has important implications for prognosis and resource allocation. A binary AKI classification can facilitate early intervention and optimize medical resource allocation for high-risk patients.

DCA results indicated that our predictive model provided better net benefits when the threshold probability was between 0.30 and 1.00. While the model demonstrated strong discrimination and calibration in the training set, its performance in the validation set was comparatively weaker. This suggests that while the model shows promise, further validation and refinement are required to enhance its generalizability and clinical applicability.

## Conclusions

The present study demonstrated that preoperative cystatin C levels serve as a predictor of postoperative AKI. Postoperative AKI was found to be associated with postoperative ACVE, ARF, and early mortality. A clinical prediction model incorporating cystatin C and other risk factors was preliminarily developed and validated.

## Dodatak

### Limitations

The data used to establish the prediction model were derived from retrospective materials, and the sample size was relatively small. While cystatin C demonstrated a strong correlation with postoperative AKI in this model, additional relevant variables should be incorporated to enhance predictive accuracy. Furthermore, external validation is necessary to confirm the model's generalizability.

### Acknowledgements

None.

### Consent to publish

The manuscript has neither been previously published nor is under consideration by any other journal. The authors have all approved the content of the paper.

### Consent to participate

Informed oral consent was obtained from all participants.

### Ethics approval

This retrospective case-control study received ethical approval from Qilu Hospital of Shandong University (KYLL-202008-017).

### Data availability statement

The data supporting the findings of this study can be obtained from the corresponding author upon request.

### Funding

None.

### Conflict of interest statement

All the authors declare that they have no conflict of interest in this work.
